# Rapid tremor migration and pore-pressure waves in subduction zones

**DOI:** 10.1038/s41467-018-05150-3

**Published:** 2018-07-24

**Authors:** Víctor M. Cruz-Atienza, Carlos Villafuerte, Harsha S. Bhat

**Affiliations:** 1 Instituto de Geofísica, Universidad Nacional Autónoma de México, 04510, Coyoacán, Mexico City, Mexico; 2 Laboratoire de Géologie, Ecole Normale Supérieur, CNRS-UMR 8538, PSL Research University, 75005, Paris, France

## Abstract

Rapid tremor migration (RTM) in subduction zones is a manifestation of complex fault-zone processes on the plate interface. Recent observations have revealed a large diversity of RTM patterns that are always associated with aseismic, shear strain at the interface. Small unstable asperities embedded in the stable shear zone are thus believed to originate tremor radiation during migration. Tectonic tremors have been recognized to occur where overpressured fluids exist. Spatial variations of fluid pressure may lead to non-linear diffusion processes with potentially large implications in tremor generation. Here, we show that pore-pressure waves are likely to exist in the plate interface, propagating with speeds and pathways similar to RTMs observed in different subduction zones including Guerrero, Mexico, where we introduce new high-resolution tremor locations and a RTM source physical model. These waves may explain the whole hierarchy of RTM patterns by producing transient reductions of the fault strength and thus secondary slip fronts triggering tremor during slow earthquakes.

## Abstract (web summary)

Rapid tremor migration in subduction zones has been associated with aseismic, shear strain at the plate interface. Here, the authors develop a physical model that shows that pore-pressure waves at the plate interface are likely to generate secondary slip fronts triggering rapid tremor migrations with speeds and pathways similar to those observed in subduction zones.

## Introduction

Slow slip events (SSE) in subduction zones^1^ have been recognized as the main driving mechanism triggering tectonic tremors (TT)^2, 3^ and low frequency earthquakes (LFE)^4^ in the vicinity of the plate interface^5^. A clear spatio-temporal correlation exists between the propagating slow slip and tremor in different subduction zones, such as Cascadia and Nankai^6, 7^. Known as episodic tremor and slip^2^ (ETS), this coupled phenomenon mostly propagates along-strike of the interface at speeds of ∼10 [km day^−1^]^6–8^. However, much faster tremor migrations have also been recognized in two other preferential directions. During the ETS propagation, localized tremor sources may migrate faster for tens of kilometers opposite to the ETS-front propagation (often along-strike) direction, with speeds from 100 to 400 km day^−1^ (i.e., 7–17 km h^−1^)^9, 10^. These rapid tremor reversals (RTR) may travel up to 45 km backward initiating in the active ETS front^11^. The second rapid migration pattern is characterized by propagating streaks at even faster speeds ranging from 25 to 150 km h^−1^ along the slip-parallel (i.e., along-dip) direction^12, 13^. Recently, high-resolution tremor locations in Cascadia and Mexico have revealed a more complex behavior of rapid tremor migrations (RTM)^14, 15^, where they also occur in directions other than the along-dip and along-strike axes, especially after the main ETS front has moved away.

Observations in different subduction zones show that tremor radiation is always accompanied by slow slip in the plate interface. This is true for the ETS main front^2, 3, 16^, and the secondary RTMs (including RTR^17^). Such observations suggest that tremor sources are triggered, in all cases, by the stress concentration over unstable asperities surrounded by the slow slipping fault^16, 18–20^. For explaining the origin of RTM, the problem then reduces to understand the conditions allowing different slip patterns during an SSE^21^. Understanding the physics of secondary slip fronts (SSF) propagating accordingly with the hierarchical RTM patterns has been a major research topic in the last years. Different works have tackled this problem based on fault constitutive models either under the rate-and-state (R&S) friction framework^22–25^ or by integrating brittle–ductile rheological considerations^26^. Although a few of these models can explain most of the RTM patterns, none of them integrates a potentially critical element that seems to be always present in the ETS environment: overpressured fluids.

Regions of the globe where ETS occurs are systematically located at depths of the plate interface (30–45 km) where there is strong evidence of overpressured fluids in the oceanic crusts (OC)^4, 27–29^. Fluid pressurization is the result of prograde metamorphic dehydration reactions producing large amounts of hydrous fluids from subducted materials^30^. Overpressured fluids have been widely evoked in the literature suggesting possible implications they may have in the generation of SSEs and tremor^29, 31, 32^. Different investigations have shown that composite faults characterized by a stable frictional matrix (i.e., dominated by velocity-strengthening (VS) constitutive parameters in the R&S friction law^33^) may undertake dynamic instabilities in embedded, velocity-weakening (VW) asperities^34, 35^, provided that the contrast in frictional strength between the matrix and the asperities is large enough. In those models, the steady-state frictional strength is proportional to the effective normal stress acting on the fault plane (given by (*a*-*b*)*σ*_e_ , where *a* and *b* are R&S parameters and *σ*_e_is the effective stress^36^), so that the contrast required to produce seismic radiation from the asperities during an SSE, and thus LFEs, is likely to be induced by spatial and/or temporal variations of pore pressure (*p*). Furthermore, experimental evidence supported by R&S friction models also show that reductions of the effective stresses during stable sliding may produce transitions from VS condition to fully unstable (VW conditions) waves raditaion^31^. Understanding how fluids may behave in the slow-earthquakes environment affecting the effective stresses could thus shed light into the actual mechanisms governing tremor generation and migration. Here, we explore a physical model capable to explain the diversity of RTM patterns as a result of transient perturbations of *p* traveling at the expected speeds and pathways along the fluid-saturated plate interface.

## Results

### Pore-pressure waves in the plate interface

**Fig. 1:**
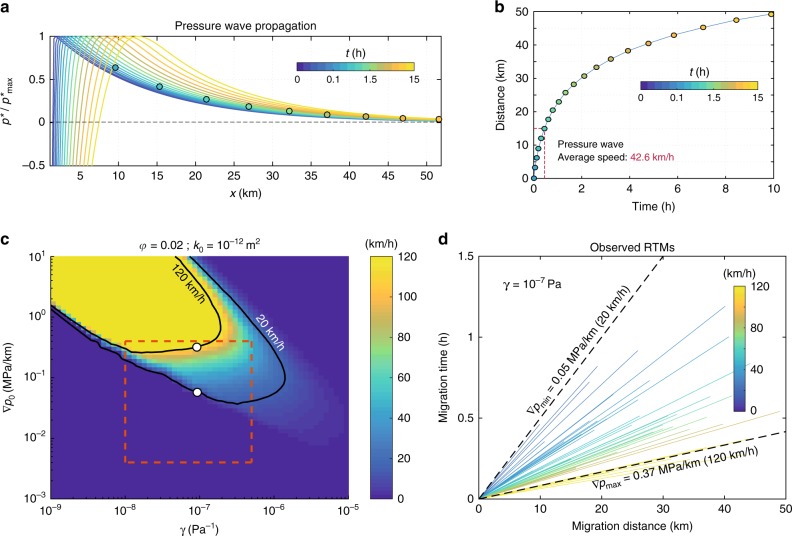
Analysis of the non-linear diffusion equation under plausible conditions for the Guerrero subduction zone. **a** Pore-pressure wave propagation predicted by Eq. (2), where p* = *p* − *p*_0_. Circles show the position of the wave front for a pore-pressure threshold of 3 kPa and color shaded the time in hours. **b** Wave front propagation (circles in panel **a**) and average speed after 15 km. **c** Parametric study of Eq. (2) in terms of wave speeds for a threshold of 3 kPa (see Supplementary Figure 3 for different permeabilities). Speed values between the black curves include those observed for RTMs in Guerrero. Red box delineates *γ* values observed in laboratory experiments^40^ and the maximum pore-pressure gradient induced by the 2006 SSE (lower limit). **d** Guerrero RTM speeds bounded by the two theoretical limits indicated with white circles in panel **c**

Fluid transport in porous media may explain seismicity migration patterns due to diffusion processes^37, 38^. The driving force for fluid diffusion is the gradient of *p* (∇*p*_0_), which can induce pressure fronts traveling with typical speeds ranging from 0.002 to 0.04 km h^−1^ in borehole injection tests^39^. Laboratory studies of rock mechanics since the late 1960s have shown that permeability (*k*) is a strongly decreasing function of the effective pressure (i.e., of *P*_*e*_ = *P*_*c*_ − *p*, where *P*_*c*_ is the confining pressure). Experiments in granite rock protoliths, fault-damaged zones, and fault-core samples under elevated confining pressures systematically show an exponential decrease of *k* as the effective pressure increases of the form^40^:

1 $$k\left( {P_e} \right) = k_0e^{\left( { - \gamma P_e} \right)},$$

where *k*_0_ is the rock permeability for *P*_*e*_ = 0, and *γ* is a constant dictating the sensitivity of permeability to a given change of *P*_*e*_. Such a nonlinear behavior of *k* leads to the general diffusion equation for fluid-saturated porous rocks given by

2 $$\frac{{\partial p}}{{\partial t}} = \frac{1}{{\phi \eta _f\left( {\beta _f + \beta _n} \right)}}\nabla \cdot \left[ {k\left( {P_e} \right)\nabla p} \right],$$

where *ϕ* is the rock porosity, *β*_*f*_ and *β*_*n*_ are the compressibilities of the fluid and the porous matrix, respectively, and *η*_*f*_ is the fluid viscosity (see Methods). Equation (2) is a nonlinear partial differential equation that admits soliton-like solutions traveling as single pore pressure waves^41^. Solitary pressure waves have been evoked to explain different geophysical phenomena^38, 42^. Conditions for these waves to exist depend on two main parameters, i.e., ∇*p*_0_ and *γ*. To explore the physical conditions where pressure waves propagate within the RTM speed range, we have developed a finite volume scheme in two dimensions for solving Eq. (2) (see Methods and Supplementary Methods). Figure 1a shows a solution of the equation where a solitary pore-pressure wave propagates for several kilometers with an average speed of 42.6 km h^−1^ (Fig. 1b). Unlike linear diffusion processes, which exhibit instantaneous pressure effects in the whole saturated medium, this perturbation travels in space while producing a transient reduction of *P*_*e*_. Behind the pressure wave, absolute *p* values decrease below their initial level inducing higher final values of *P*_*e*_.

Borehole measurements and indirect estimates based on temperature and flow meters logs in different subducted OCs indicate permeability values from 10^−16^ and up to 10^−11^ [m^2^]^43^. As the confining pressure increases down into depths where most SSEs and TTs take place (30–45 km), porosity (*ϕ*) in the OC and the serpentinized mantle wedge decreases down to 2–4.5%^28^, with estimated permeabilities ranging between 10^−17^ and 10^−22^ [m^2^]^28, 29^. However, large-scale stepwise changes in permeability at the onset of slip are produced due to the strong fault-zone-cracks aperture dependence of permeability, where earthquake-induced increases in rock mass permeability may reach values of 10^−13^ [m^2^]^44^. The aperture of fault-zone cracks or veins due to tectonic processes is supported by geological evidence in exhumed subduction-zone rocks and can vary from 10^−6^ to 10^−3^ m^45–47^, which implies permeability values of 10^−13^ < *k* < 10^−8^ m^2^ across fault-zone fluid pathways from the relationship *k* = *b*^2^/12, where *b* is the vein aperture in meters^48^. Overpressured fluids subject to the plate-motion strain field (e.g., SSEs) may also increase *k* up to several orders of magnitude due to hydrofracturing processes and preexistent fault-zone cracks unclamping that may produce prominent fluid-migration channel networks^44, 47, 49^. Observations in exhumed rocks of plate-interface shear zones^45^ and fluid-flow experiments in sheared serpentinite rocks^50^ also reveal a large anisotropy of permeability that enhances fluid transport along the plate-interface direction within the fault damage-zone. Since *k* in that direction is about two orders of magnitude larger than its value along the interface-perpendicular axis, we neglect fluid transport in the later direction within the RTMs time scale. In the following, we thus assume that diffusion takes place only in the damage zone along the two dimensions that are parallel to the plate interface.

We carried out a parametric study of Eq. (2) to identify the physical conditions leading to pore-pressure waves traveling with the RTM speeds considering laboratory-observed and estimated values for *k*_0_, *γ*, *ϕ*, and ∇*p*_0_ (see Methods). For each combination of these parameters, we solved Eq. (2) assuming values for the remaining constants shown in Table 1, and searched for propagating pressure waves in the simulation domain. In case a wave was detected, we quantified its average propagation speed along 15 km by tracking the front where *p* reaches a threshold of 3 kPa (circles in Fig. 1a, b), value that implies a similar drop of *P*_*e*_ and thus of the fault strength. The threshold corresponds to a representative mean value of global TT sensitivity to terrestrial tides^19^.

**Table 1:** Constant parameters assumed for all simulations presented in this work

Model parameter	Value
Fluid viscosity (*η*)	9.54 × 10^‒5^ Pa s
Porous matrix compressibility (*β*_*n*_)	6.5 × 10^‒10^ Pa
Fluid compressibility (*β*_*f*_)	6.4 × 10^‒10^ Pa

**Fig. 2:**
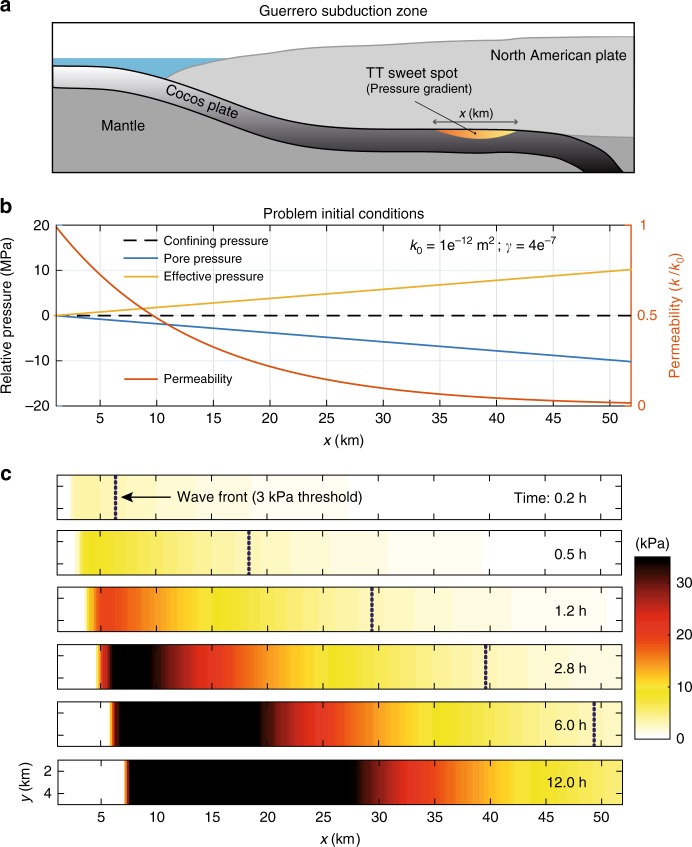
Downdip pore-pressure wave solution under plausible conditions for the Guerrero subduction zone. **a** Cartoon showing the geometry of the oceanic Cocos plate under the continent. The gradient of gray colors illustrates the lithostatic pressure in the subducted slab, while color gradient illustrates local pore pressure changes in the sweet spot. **b** Initial conditions and parameter values for the simulation presented in Fig. 1a, b. **c** Resulting pore-pressure wave propagation in the 2D domain. The dashed black line depicts the wave front for a pressure threshold of 3 kPa

Figure 1c shows, for *k*_0_ = 1 × 10^−12^ m^2^ and *ϕ* = 2%, the propagation speeds of pressure waves for different combinations of ∇*p*_0_ and *γ*. Predicted speeds range from 20 to 120 km h^−1^ within the model-space lying between the black contours. As a reference, the red square delineates the range of *γ* values measured in the laboratory, ∇*p*_0 _corresponding to those induced by the 2006 SSE in Guerrero (see next section) as the lower limit, and an arbitrary upper limit. The parametric analysis revealed that soliton-like solutions dominate the pore-pressure evolution provided that there is also a preexisting gradient of *P*_*e*_. Following Eq. (1), spatial variations of *P*_*e*_ set up heterogeneous initial values of *k*. Figure 2b illustrates this condition, where *k* is significantly larger in the region where *p* is close to the confining pressure, *P*_*c*_, that we have assumed constant here because of the horizontal configuration of the Cocos plate in Guerrero (Fig. 2a, see next section), but that may also change in space (e.g., with depth) producing similar results (see Supplementary Figure 1 for a Cascadia-like example). In all cases, solitary waves propagate from higher to lower pore-pressure regions as shown, for instance, in Figs. 1a and 2c.

### Rapid tremor migration in Guerrero, Mexico

**Fig. 3:**
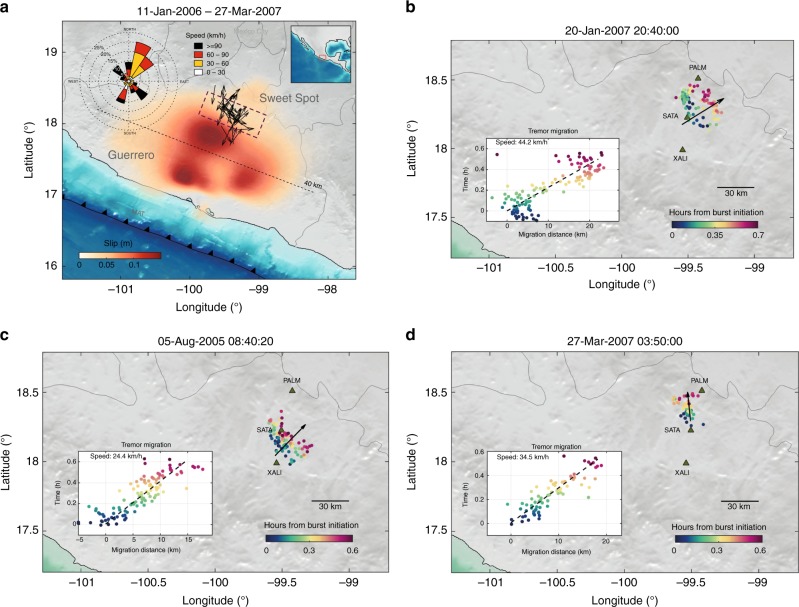
Rapid tremor migrations observed in Guerrero. **a** Migration direction and position (black arrows) of the 54 RTMs determined from seismic records in the MASE array of stations^58^ using the TREP method^57^. The wind-rose histogram shows the directions and speeds of the whole RTM catalog. As a reference, color shade shows the final slip of the 2006 SSE^55^. The dashed line indicates where the subducted Cocos plate becomes horizontal at 40 km depth, and the wine rectangle the positions of the sweet spot. **b**–**d** Examples of RTMs for 1-min moving windows with 20 s overlap. Hypocentral projections onto the migration directions (black arrows) are shown in the insets, where migration speeds are reported (see Supplementary Figure 2 for more RTM examples). The basemaps were created using SRTM15+ data

Similarly to other subduction zones, such as Nankai, Cascadia, Alaska, Costa Rica, and Hikurangi, sinking of the Cocos plate underneath the North American plate in central Mexico produces a diversity of slow earthquakes. Among them, the largest SSEs in the globe taking place in Guerrero every 3.5 years with moment magnitude around 7.5^51^. Additionally, TT^52^, LFE^53^, and very low frequency earthquakes^54^ have also been observed in that province. Figure 3a shows the final slip produced by a long-term SSE occurred between March and December 2006^55^, where the dashed line indicates the distance from the trench where the plate interface becomes horizontal at 40 km depth^56^. Here, we carefully analyzed a high-resolution TT catalog recently introduced^20^ to search for RTMs in Guerrero. TT hypocentral locations have 5 km uncertainties in the three components and were obtained using the tremor energy and polarization (TREP) method^57^ from broadband seismic data recorded in the MASE array^58^ between January 2005 and March 2007. Most of hypocenters lie close to the horizontal plate interface^20^, so we only considered TT sources between 40 and 45 km depth for the analysis. We found 54 RTMs in the catalog; each one associated with an individual tremor burst. Figure 3b–d and Supplementary Figure 2 show some representative examples of these rapid migration patterns.

**Fig. 4:**
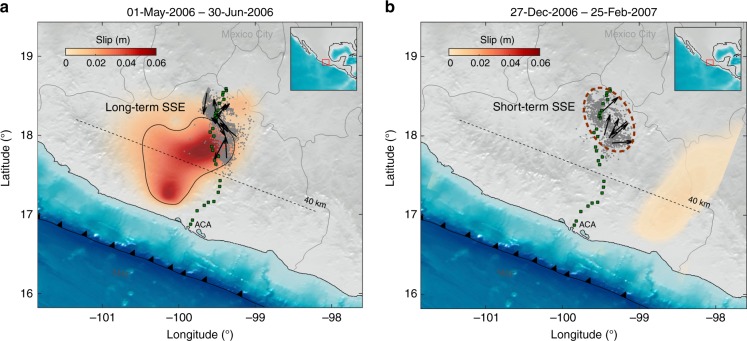
Slip increments of the 2006 SSE for the periods indicated on top of each panel. Tremor epicenters and RTMs associated with each period are shown in gray dots and black arrows, respectively. While TTs and RTMs shown in panel **a** correspond to those occurred during a stage of the long-term SSE, panel **b** shows tremors associated with a slip reactivation (i.e., a short-term SSE, wine dotted line), months later the long-term SSE has moved away from the region. The basemaps were created using SRTM15+ data

Projected horizontal locations into the migration directions (black arrows) allowed us to estimate the tremor migration speeds and distances (insets). Migration directions correspond to the azimuth that maximizes the speed after projection. The wind-rose diagram of Fig. 3a summarizes the whole RTM directions and speeds, from which 26 RTMs occurred during the 2006 long-term SSE. The remaining 28 migrations occurred during inter-SSE tremor bursts associated with short-term slow earthquakes^15, 53^. For example, Fig. 4a shows the RTMs found between May and June during the long-term 2006 SSE, while Fig. 4b shows the RTMs produced by a short-term SSE in January 2007, months later the long-term SSE has passed through that region. Almost 90% of the RTMs are located close to the downdip limit of the long-term slow slip, in the so-called sweet spot^59^ (Fig. 3a), where tremor activity dominates in the region. There is a preferential trench-perpendicular (i.e., slip-parallel) migration pattern with speeds ranging from 30 to 90 km h^−1^, which is consistent with recent estimates in Guerrero^15, 53^ and with the tremor streaks found in Nankai and Cascadia. There are also RTMs in the along-strike (and updip) directions with even faster speeds (60–120 km h^−1^), which may probably correspond to RTRs.

Figure 1d presents the whole RTM catalog (color lines) bounded by lower and upper speed limits predicted by Eq. (2) for pore-pressure gradients of 0.05 and 0.37 MPa km^−1^ (black dashed lines). These bounding models correspond to the white circles in Fig. 1c, for which values of *k*_0_, *ϕ*, and *γ* are 10^−12^ m^2^, 2%, and 10^−7^ Pa^−1^, respectively. Other physical conditions may also produce pressure waves with speeds similar to those of the RTM catalog. Supplementary Figure 3 shows the parametric results for three other values of *k*_0_, where pressure waves barely propagate for *k*_0_ equal or less than 10^−14^ m^2^ and very high-pressure gradients (i.e., ∇*p*_0_ > 4 MPa km^−1^).

Migration speeds for different porosities (1 and 2%) and wave front thresholds (1–10 kPa) are also presented in Supplementary Figure 4, from where we concluded that plausible ranges for the model parameters producing pressure waves in the plate interface with speeds between 1 and 200 km h^−1^ are those reported in Table 2. Note that these speed limits enclose not only our RTMs in Guerrero but also observed values in other subduction zones worldwide.

**Table 2:** Ranges of the model parameters where soliton pore-pressure waves may propagate with speeds between 1 and 200 km h^−1^ under plausible conditions for subduction zones where RTMs are observed

Model parameter	Range of values
Permeability (*k*_0_)	10^−14^–10^−11^ m^2^
Porosity (*ϕ*)	0.01–0.02
Pore pressure gradient (∇*p*_0_)	0.02–5 MPa km^−1^
Sensitivity (*γ*)	1 × 10^−8^–5 × 10^−7^ Pa^−1^
Pore pressures threshold	1–10 kPa

### SSFs and RTMs associated with pore-pressure waves

Recent high-resolution TT locations have revealed unprecedented observations of the RTM spatio-temporal behaviors in Cascadia and Mexico^14, 15^. Rapid migration patterns evolve as the main slow-slip front passes through the tremor region. Initially, RTMs are more frequent, mostly follow the main SSE front geometry and have larger migration speeds^15^. This can partly be appreciated in Fig. 4a, where most RTMs are parallel to the SSE front. As the front moves away, RTMs recurrence times become longer and tidally modulated, while their migration speeds decrease^15, 19, 60^. Figure 4b shows, for instance, that once the SSE front is far away, the RTMs preferential direction is reoriented along dip.

Fault-zone pressure waves may control the plate-interface effective normal stresses (*σ*_*e*_) and thus the fault strength that, as mentioned earlier, has strong implications in the slip velocity under the R&S friction law. In the condition of variable effective stress, the fault state variable changes according to the jump of *σ*_*e*_^33^. For constant shear (*τ*) and normal (*σ*) tractions, a change in the effective stress from the initial, $\sigma _e^i = \left( {\sigma - p_i} \right)$, to the final, $\sigma _e^f = ( {\sigma - p_f} )$, states, produces a response in the slip velocity given by^31^

3 $$\frac{{v_f}}{{v_i}} = \left( {\frac{{\sigma _e^{\,f}}}{{\sigma _e^i}}} \right)^{\left( {\alpha /a} \right)}{\mathrm{exp}}\left[ {\frac{\tau }{a}\left( {\frac{1}{{\sigma _e^{\,f}}} - \frac{1}{{\sigma _e^i}}} \right)} \right],$$

where *α* is about 0.2 for slow slip rates, and *v*_*i*_ and *v*_*f*_ are the initial and final slip velocities, respectively, due to the change in pore pressure Δ*p* = *p*_*f*_ − *p*_*i*_. For tractions *τ* = 8.5 MPa and $\sigma = \sigma _e^i = 2$ MPa, which are reasonable values for the SSE plate interface environment^27, 45^, small values of Δ*p* (i.e., between 1 and 20 kPa, that correspond to the typical stress perturbation of a pressure wave as shown in Fig. 2c) may produce *v*_*f*_ up to two orders of magnitude higher than *v*_*i*_ (Supplementary Figure 5). Such slip accelerations are consistent with estimates for the slip velocity of SSF as compared with the typical velocities of the SSE main fronts^17, 21^. The effective-stress transient reduction induced by pressure waves may thus potentially lead to SSFs triggering RTMs via the stress transfer from the stable fault matrix to embedded unstable asperities^20, 25, 26, 35^. However, a critical condition for these waves is the existence of pore-pressure gradients in the fault zone where the SSEs take place. Pressure gradients in tremor zones may result from dilation changes induced by ongoing or past SSEs, localized dehydration reactions in the subducted slab, variations in the geometry of the slab and/or the plate interface, or different combinations of these mechanisms.

Our observations of RTMs in Guerrero show a dominant migration direction away from the trench (Fig. 3a). We thus explored whether the residual (static) trench-perpendicular pore-pressure gradient induced by the 2006 SSE in the OC could explain this observation. However, as shown in Supplementary Figure 6, although *p* decreases in the sweet spot with distance from the trench, the maximum gradient (2 kPa km^−1^) is significantly smaller (about one order of magnitude) than those required to produce pressure waves with the expected speeds (Table 2). Though the residual strain field from cumulative SSEs may lead to larger ∇*p*_0_, an additional preexistent gradient seems necessary in Guerrero to meet the physical conditions producing such waves.

**Fig. 5:**
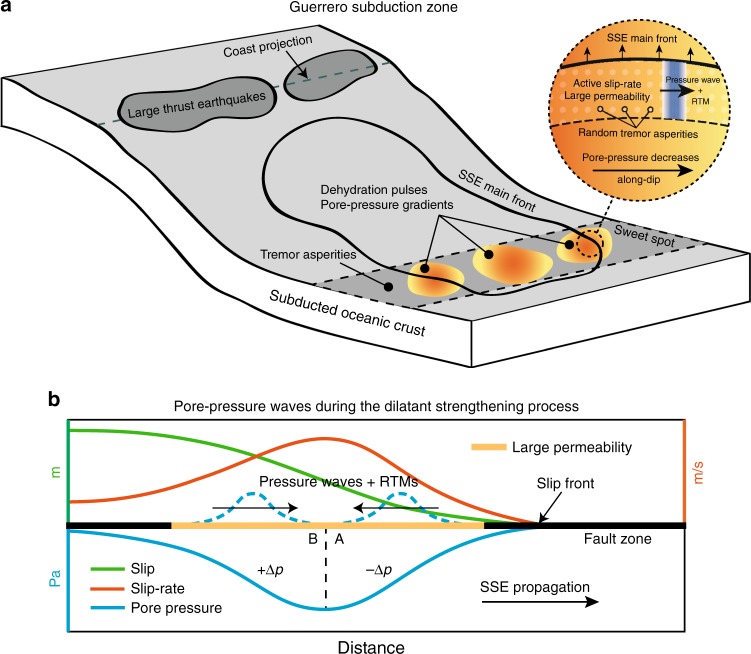
Cartoons illustrating the mechanisms we propose to explain the existence of pore-pressure waves in the plate interface and the associated generation of RTMs. **a** The Cocos plate interface during a SSE experiences a RTM due to the propagation of a pressure wave and thus a SSF, which is driven by a preexistent ∇*p*_0_ associated with a localized dehydration pulse in the oceanic crust. A highly permeable zone associated with the active SSE front channelizes the wave. This mechanism is likely to explain RTMs following the SSE front (e.g., tremor streaks). **b** Plate interface conditions set by the dilatant strengthening mechanism during a SSE. Two opposite pore-pressure gradients are generated in the SSE front-perpendicular direction where permeability is increased. This mechanism is likely to explain both the rapid tremor reversals (RTRs) and RTMs in the SSE propagation direction

Prominent gradients of pore pressure (as large as 3 MPa km^−1^) in subducted OCs have been inferred from tomographic P-wave imaging^61^ and thermomechanical slab modeling^62^. Those preexistent gradients represent the pumping force driving fluids into the mantle and through in-slab normal faults. Metamorphic dehydration reactions in the OC depend on both, local pressure/temperature conditions, and the composition of the subducted materials. In Guerrero, tremor activity concentrates in the sweet spot over separated along-strike patches^54^. This observation suggests that mechanical and/or fluid pressure conditions in those patches are different from the neighboring segments, as supported by mineralogical phase diagrams and thermal modeling of the subducted Cocos plate, which indicate that the largest dehydration pulse takes place in the top of the slab right in the sweet spot^63^. These arguments are certainly valid for other subduction zones, where overpressure fluids should also be confined in space^47^. As we move from the optimal dehydration zones, pore pressure should decrease due to diffusion processes and lower fluid production rates (Fig. 5a). This mechanism seems a reasonable candidate for setting preexistent conditions promoting pressure waves in Guerrero and thus SSFs in the down-dip direction. Since most tremor migrations are parallel to the SSEs main-front^15^, there should be a dominant pore-pressure reduction across the sweet spot (inset of Fig. 5a) with distance from the trench.

**Fig. 6:**
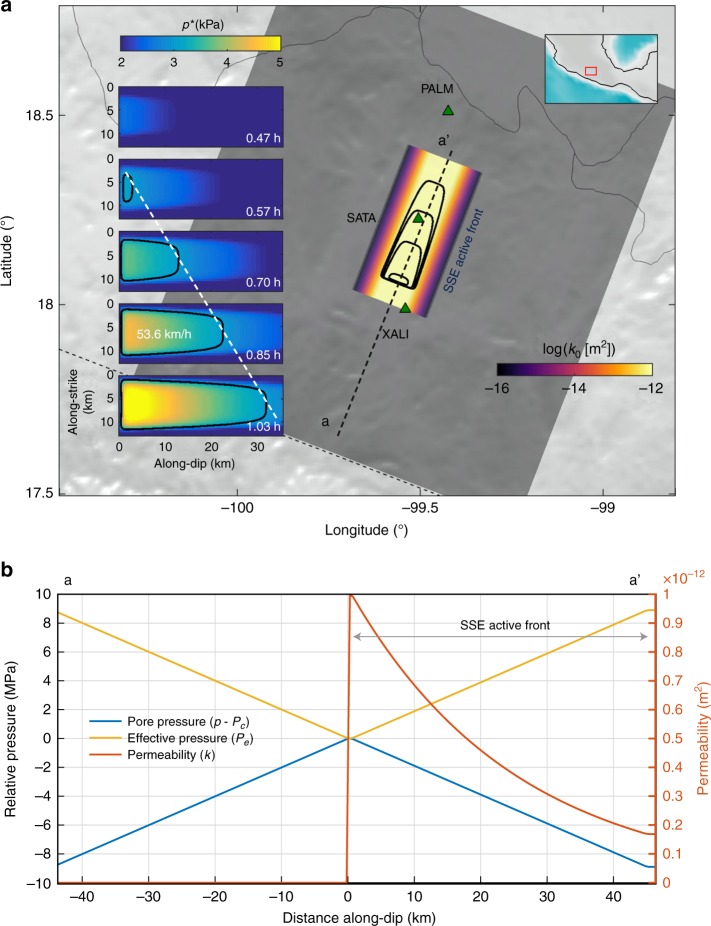
Two-dimensional pore-pressure wave simulation in Guerrero. In this model, permeability *k*_0_ gradually increases (black–yellow shade in panel **a**) inside an elongated trench-perpendicular region that we assume corresponds to the most active SSE front (i.e., where slip-rate is significantly large). The blue-yellow shade in panel **a** shows the evolution of pore pressure (where p* = *p* − *p*), while the black contours indicate the position of the wave-front for a pressure threshold of 3 kPa. Compare with Fig. 3. Panel **b** shows the initial conditions for the simulation along the cross-section a–a′ depicted with a dashed black line in panel **a**. The basemap was created using SRTM15+ data

When the SSE main front passes through a region with high-enough ∇*p* (approximately larger than 20 kPa km^−1^, Table 2) and nearly zero effective pressure, the permeability increases along the front channelizing the pathway for pressure waves and RTMs. Large localized increments of *k* (i.e., of several orders of magnitude) due to shear slip in the fault zone have been recognized in borehole injection tests^44^ and suggested to have implications for tremor generation^15^. The inset of Fig. 5a illustrates this, where pressure waves propagate driven by the *p* gradient following the highly-permeable SSE front. We numerically explored this idea by solving Eq. (2) over a plane laying 1 km below the plate interface taking, as initial conditions for *p*, the final distribution of the pore-pressure induced by the 2006 SSE in the sweet spot (Supplementary Figure 6) plus a constant preexistent gradient (along-dip reduction of *p*) of 20 kPa km^−1^. The model assumes that permeability is high in the slow-slip active front that we approximated, according to previous authors, as a 5 km width trench-perpendicular region^15^ (Fig. 6a). In this region, *k*_0_ gradually increases from 10^−16^ m^2^ in its surroundings (i.e., outside the front) to 1 × 10^−12^ m^2^ in the middle of the region. A cross-section of the problem initial conditions in terms of *p*, *P*_*e*_, and *k* are shown in Fig. 6b. The resulting pore-pressure evolution reveals a solitary wave propagating with an average speed of 50 km h^−1^ and down-dip direction similar to those observed for the dominant RTMs in Guerrero (compare with Fig. 3b–d).

## Discussion

While RTMs along the main SSE front are naturally associated with the most-active slip region, understanding tremor migrations in the front-perpendicular direction such as the RTRs is not as intuitive. These migrations, which are less frequent, can happen either in the forward or backward SSE propagation direction^9, 10, 14^. In general, RTM speeds decrease and become less frequent as the main SSE front moves away from the tremor region. This observation can be explained by a drop of pore-pressure in the active SSE front due to the host-rock transient dilation induced by the shear slip^15^. Such mechanism, known as the dilatant strengthening process^64–66^, increases the effective normal stresses in the shear zone producing stable slip (i.e., slow earthquakes) even in VW fault regions^32^. An outstanding consequence of this process is the generation of pore-pressure gradients behind the propagating SSE front. Figure 5b illustrates this condition, where two opposite, front-perpendicular gradients are set as a consequence of the transient dilation^32^. In region A, *p* decreases with distance from the front as the slip-rate develops to reach its maximum value. Further behind, in region B, pore-pressure recovers as the rock returns to its original undilated state producing a gradual increase of *p*. Numerical investigations of the dilatant strengthening process show that the associated ∇*p* may be of the order of 80 [kPa km^−1^]^66^. Although it is difficult to quantify from the available results whether the corresponding *P*_*e*_ meets the conditions producing pressure waves, since gradients between 20 and 5000 kPa km^−1^ induce waves propagating with the expected RTM speeds (Table 2), the dilatant strengthening process may potentially set suitable conditions for the existence of pressure pulses. The pore-pressure gradient in region A could thus potentially be the driving force for pulses (and the associated SSFs) triggering RTRs, while the gradient in region B, could promote RTMs in the opposite forward direction, as observed in nature. Since preexistent gradients in the OC may be highly variable in space it is possible that, in some regions, the dominant gradients are those induced by the dilatant strengthening process thus promoting pressure pulses triggering RTRs and forward RTMs. In Guerrero, transient reductions of the crustal wave speed have been observed during SSEs^67^. This extraordinary observation, which may be explained by the non-linear dilatant-strengthening response of the deep crust^68^, suggest that some RTMs we reported for the 2006 SSE could be produced by the physical mechanism illustrated in Fig. 5b.

In addition to the fault-zone dilatant process that seems to be a plausible mechanism explaining the speed and recurrence of RTMs as the SSE propagates^15^, pore-pressure waves also produce a trailing drop of *p* as they sweep the RTM pathway (Fig. 1a). The result of this process is a reduction of ∇*p*_0_ in the tremor zone. Figure 1c shows that migration speed of pressure waves decreases with reductions of ∇*p*_0_ (e.g., along a vertical path joining both white circles). Thus, successive waves, propagating across similar paths, will slow down with recurrence time. Besides this, since overpressured fault spots where pressure waves are expected to born would also experience an increment of *P*_*e*_, the origin time of the next wave would be delayed (i.e., the recurrence time would increase) as the SSE strain field evolves in a similar way RTM behave. The pressure gradient reduction depends on the healing and resealing of the fault zone behind the associated SSF as *P*_*e*_ grows during the trailing drop of *p*. Future work should certainly integrate these mechanical processes into the model to identify other factors controlling the evolving nature of RTMs during an SSE episode.

Our modeling results also suggest another explanation for the RTM speed reduction with recurrence time. The parametric study we carried out assumed, for each parameters combination, the same frictional strength for tremor asperities (Fig. 1c and Supplementary Figure 6). However, the larger the strength, the slower is the pressure front. This can be seen in Supplementary Figure 4 (solid curves), where the front speed slows down by a factor of three for strength increments of the same order (i.e., from 1 to 5 kPa). In a fault where tremor asperities with different strengths are randomly distributed, the front of a pressure wave triggering weaker asperities will travel faster and manifest earlier than the slower trailing front triggering stronger asperities. This mechanism would translate into successive RTMs with decreasing speeds.

Our observations of RTMs in Guerrero (Fig. 3 and Supplementary Figure 2) reveal that tremor migrates as 5–10 km width source packages, which is consistent with results using different TT location methods in Guerrero and Cascadia^14, 15, 21^. One possible explanation of this migration pattern is that the active slip region associated with the SSFs describes a pulse-like perturbation responding to some self-healing frictional process. Our pore-pressure wave model also predicts propagating pulse-like perturbations of the fault-zone effective pressure (e.g., Figs. 1a and 2c). Nevertheless, their widths and migration speeds are significantly larger and smaller, respectively, than those observed for RTMs. The actual fault response to such pressure transients (e.g., its slip rate) may not be as intuitive though. In other terms, the associated SSF may not necessarily mimic the evolution of *p* along the fault that, in a R&S friction model coupled with a time varying evolution of *p*, strongly depends on the temporal derivative of *p* (i.e., on *dp*/*dt*). Modeling results for fluid injection tests next to faults subject to such friction law show that slip instabilities occur, in fact, where d*p*⁄d*t* is close to its maximum value and *p* grows monotonically^69^. For our pore-pressure wave model and its implications in tremor generation, this means that the associated SSFs could take place well before reaching the maximum value of *p* in the fault zone, so that the pressure pulse does not necessarily depicts the shape of the associated SSF, which may be significantly narrower and have faster migration speed. This, of course, deserves further analysis of the non-linear diffusion problem (Eq. (2)) coupled with R&S friction laws in the fault plane, which goes beyond the scope of this work but represents the current direction of our investigations.

There also exists the possibility that the RTM pulse width may be significantly narrower than the SSF. This can be seen considering that tremor asperities radiating waves during the same RTM are expected to have similar strengths^15, 19^. As the SSF propagates and successively surrounds the (locked) tremor asperities, it charges the asperities until they break at the moment their instability condition is reached no matter that the slip front continues developing behind the broken asperities^20^. This simple principle suggests that pressure pulses, which presumably induce SSFs with even wider active regions than the RTM front, are likely to trigger pulse-like tremor migrations as observed in Guerrero and other places. In other words, pressure pulses should not be as narrow as the RTM fronts to be consistent with observations.

In summary, observational and theoretical evidence worldwide strongly suggest that TT is always triggered by small unstable asperities embedded in a slow-sliding (stable) fault zone saturated with overpressures fluids. Such mechanism is also valid to explain RTMs due to much faster, SSFs propagating along the fault. Here we show that pore-pressure waves are likely to propagate across the plate-interface fault zone with the expected RTM speeds and pathways provided that moderate pore pressure gradients ($\gtrsim 20$ kPa km^−1^) exist in the SSE region. Those gradients can be generated either by preexisting and localized dehydration processes in the OC, variations of the interface geometry and the SSE-induced strain field (i.e., through the dilatant strengthening processes). During propagation, pore pressure waves produce transient reductions of the effective fault-normal stresses that may lead to SSFs triggering tremor via the stress transfer into the asperities. Although this idea should be rigorously explored by means of a R&S friction model coupled with the non-linear fluid-diffusion equation, rapid pressure waves in that framework seem a plausible mechanism capable to explain the hierarchical diversity of RTMs patterns observed in different subduction zones including Guerrero, Mexico, where cutting-edge seismogeodetic instrumentation, offshore and onshore, has been recently deployed to improve the characterization of slow earthquakes and constrain our RTM model^70^. The model we propose opens a new area of research that may help to better understand the fault system in different geophysical conditions such as volcanic systems, geothermal fields, and production wells with induced seismicity, where the seismic hazard is high and should be assessed by means of physics-based modeling considerations.

## Methods

### Non-linear diffusion equation with variable permeability

The spatio-temporal evolution of pore pressure in fluid-saturated media is given by the hydraulic diffusion equation. To deduce this equation for a given function of permeability such as Eq. (1), let us start by the equation of mass conservation,

4 $$\frac{{\partial m}}{{\partial t}} + \nabla \cdot \underline q \left( {\underline x } \right) = 0,$$

where *m* represents the fluid mass for unit volume of porous medium, $\underline q (\underline x )$ is the fluid-discharge velocity vector, *t* is time, and $\underline x$ is a general position vector. From Darcy’s law, the fluid discharge may be expressed as

5 $$\underline q \left( {\underline x } \right) = - \frac{{\rho _f}}{{\eta _f}}k\left( {\underline x } \right)\nabla p\left( {\underline x } \right),$$

where *ρ*_*f*_ and $\eta _f$ are the fluid density and viscosity, respectively, $k(\underline x )$ is a general function of permeability, and *p* is the pore pressure. Given that the total fluid mass can be expressed in terms of porosity*, ϕ*, and the fluid density as $m = \phi \rho _f$, then

6 $$\frac{{\partial m}}{{\partial t}} = \phi \frac{{\partial \rho _f}}{{\partial t}} + \rho _f\frac{{\partial \phi }}{{\partial t}},$$

Ignoring thermal and anelastic effects, the temporal derivatives of the right-hand term are given by

7 $$\frac{{\partial \rho _f}}{{\partial t}} = \rho _f\beta _f\frac{{\partial p\left( {\underline x } \right)}}{{\partial t}}$$

and

8 $$\frac{{\partial \varphi }}{{\partial t}} = \phi \beta _n\frac{{\partial p\left( {\underline x } \right)}}{{\partial t}},$$

where *β*_*f*_ and *β*_*n*_ are the fluid and porous matrix compressibilities, respectively. Substituting (7) and (8) into Eq. (6), we have

9 $$\frac{{\partial m}}{{\partial t}} = \phi \rho _f\left( {\beta _n + \beta _f} \right)\frac{{\partial p\left( {\underline x } \right)}}{{\partial t}}.$$

Inputting (5) and (9) into Eq. (4), we finally obtain the diffusion equation for a general function of permeability

10 $$\frac{{\partial p\left( {\underline x } \right)}}{{\partial t}} = \zeta \nabla \cdot \left[ {k\left( {\underline x } \right)\nabla p\left( {\underline x } \right)} \right]$$

where

$$\zeta = \frac{1}{{\phi \eta _f\left( {\beta _f + \beta _n} \right)}}.$$

If permeability varies with effective pressure (i.e., with $P_e\left( {\underline x } \right) = P_c\left( {\underline x } \right) - p\left( {\underline x } \right)$) following the exponential form given by Eq. (1)^40^, then Eq. (10) states the non-linear problem we are interested in this investigation:

11 $$\frac{{\partial p\left( {\underline x } \right)}}{{\partial t}} = \zeta \nabla \cdot \left[ {k\left( {P_c\left( {\underline x } \right) - p\left( {\underline x } \right)} \right)\nabla p\left( {\underline x } \right)} \right].$$

As mentioned earlier in the paper, besides predicting the linear fluid diffusion, this partial differential equation admits soliton-like solutions traveling as single pore pressure waves under certain conditions^41^.

### 2D finite volume method for solving the non-linear diffusion equation

To solve the nonlinear partial differential equation (Eq. (11)) in two dimensions (2D) we applied the finite volume (FV) method. We choose this method because it is conservative and stable when solving diffusion problems, which is an important property to study pressure waves propagation avoiding numerical dispersion. Our 2D FV method decomposes the domain into *N* × *M* rectangular elements (or control volumes) in the *x*–*z* plane with sizes $\Delta x \times \Delta z$, as illustrated in Supplementary Figure 7.

Assuming the general case for anisotropic and variable permeability (i.e., $k(x,z,t)$), we have that

12 $$\underline {\underline k } = \left[ {\begin{array}{*{20}{c}} {k_{xx}} & {k_{xz}} \\ {k_{zx}} & {k_{zz}} \end{array}} \right].$$

Since the fluid diffusivity is given by $\underline {\underline K } = \zeta \underline {\underline k }$, then Eq. (11) may be written as

13 $$\frac{{\partial p}}{{\partial t}} = \nabla \cdot \left[ {\underline {\underline K } \cdot \nabla p} \right],$$

where the pore pressure, *p*(*x*, *z*, *t*), is also a function of space and time. Developing Eq. (13) and integrating both terms in a given volume (Δ*V*),

14 $${\int_{{\mathrm{\Delta }}V}} \frac{{\partial p}}{{\partial t}}{\mathrm d}V = {\int_{{\mathrm{\Delta }}V}} \frac{\partial }{{\partial x}}\left[ {K_{xx}\frac{{\partial p}}{{\partial x}} + K_{xz}\frac{{\partial p}}{{\partial z}}} \right]{\mathrm d}V + {\int_{{\mathrm{\Delta }}V}} \frac{\partial }{{\partial z}}\left[ {K_{zx}\frac{{\partial p}}{{\partial x}} + K_{zz}\frac{{\partial p}}{{\partial z}}} \right]{\mathrm d}V.$$

Since *K*_*xx*_ is constant within a control volume and considering the indexes shown in Supplementary Figure 7, the first integral of the right-hand term may be expressed as

15 $$\begin{array}{*{20}{l}} {{\int_{\Delta V}} \frac{\partial }{{\partial x}}\left[ {K_{xx}\frac{{\partial p}}{{\partial x}}} \right]{\mathrm d}V} \hfill & = \hfill & {{\int_{z_{j - 1/2}}^{z_{j + 1/2}}} {\int_{x_{i - 1/2}}^{x_{i + 1/2}}} \frac{\partial }{{\partial x}}\left[ {K_{xx}\frac{{\partial p}}{{\partial x}}} \right]{\mathrm d}x{\mathrm d}z} \hfill \\ {} \hfill & = \hfill & {{\int_{z_{j - 1/2}}^{z_{j + 1/2}}} \left[ {K_{xx}\frac{{\partial p}}{{\partial x}}} \right]|_{x_{i - 1/2}}^{x_{i + 1/2}}{\mathrm d}z} \hfill \\ {} \hfill & = \hfill & {{\int_{z_{j - 1/2}}^{z_{j + 1/2}}} \left[ {K_{xx_{i + 1/2}}\left( {\frac{{\partial p}}{{\partial x}}} \right)_{i + 1/2} -\, K_{xx_{i - 1/2}}\left( {\frac{{\partial p}}{{\partial x}}} \right)_{i - 1/2}} \right]{\mathrm d}z} \hfill \\ {} \hfill & = \hfill & {\Delta zK_{xx_{i + 1/2}}\left( {\frac{{\partial p}}{{\partial x}}} \right)_{i + 1/2} - \,\Delta zK_{xx_{i - 1/2}}(\frac{{\partial p}}{{\partial x}})_{i - 1/2}.} \hfill \end{array}$$

Following the same procedure for the remaining integrals of the right-hand term of Eq. (14), we have that

16 $${\int_{{\mathrm{\Delta }}V}} \frac{\partial }{{\partial x}}\left[ {K_{xz}\frac{{\partial p}}{{\partial z}}} \right]{\mathrm d}V = {\mathrm{\Delta }}zK_{xz_{i + 1/2}}\left( {\frac{{\partial p}}{{\partial z}}} \right)_{i + 1/2} - \,\Delta zK_{xz_{i - 1/2}}\left( {\frac{{\partial p}}{{\partial z}}} \right)_{i - 1/2}$$

17 $${\int_{{\mathrm{\Delta }}V}} \frac{\partial }{{\partial z}}\left[ {K_{zx}\frac{{\partial p}}{{\partial x}}} \right]{\mathrm d}V = {\mathrm{\Delta }}xK_{zx_{j + 1/2}}\left( {\frac{{\partial p}}{{\partial x}}} \right)_{j + 1/2} -\, {\mathrm{\Delta }}xK_{zx_{j - 1/2}}\left( {\frac{{\partial p}}{{\partial x}}} \right)_{j - 1/2}$$

18 $${\int_{{\mathrm{\Delta }}V}} \frac{\partial }{{\partial z}}\left[ {K_{zz}\frac{{\partial p}}{{\partial z}}} \right]{\mathrm d}V = {\mathrm{\Delta }}xK_{zz_{j + 1/2}}\left( {\frac{{\partial p}}{{\partial z}}} \right)_{j + 1/2} -\, {\mathrm{\Delta }}xK_{zz_{j - 1/2}}\left( {\frac{{\partial p}}{{\partial z}}} \right)_{j - 1/2}.$$

We approximate the spatial derivatives of pore pressure along the element boundaries using a second-order finite difference scheme, so that

19 $$\begin{array}{l}\left( {\frac{{\partial p}}{{\partial x}}} \right)_{i + 1/2} \approx \frac{{p_{i + 1,j} - p_{i,j}}}{{{\mathrm{\Delta }}x}}\\ \left( {\frac{{\partial p}}{{\partial z}}} \right)_{i + 1/2} \approx \frac{{p_{i + 1/2,j + 1/2} - p_{i + 1/2,j - 1/2}}}{{{\mathrm{\Delta }}z}}\\ \left( {\frac{{\partial p}}{{\partial x}}} \right)_{j + 1/2} \approx \frac{{p_{i + 1/2,j + 1/2} - p_{i - 1/2,j + 1/2}}}{{{\mathrm{\Delta }}x}}\\ \left( {\frac{{\partial p}}{{\partial x}}} \right)_{i - 1/2} \approx \frac{{p_{i,j} - p_{i - 1,j}}}{{{\mathrm{\Delta }}x}}\\ \left( {\frac{{\partial p}}{{\partial z}}} \right)_{i - 1/2} \approx \frac{{p_{i - 1/2,j + 1/2} - p_{i - 1/2,j - 1/2}}}{{{\mathrm{\Delta }}z}}\\ \left( {\frac{{\partial p}}{{\partial x}}} \right)_{j - 1/2} \approx \frac{{p_{i + 1/2,j - 1/2} - p_{i - 1/2,j + 1/2}}}{{{\mathrm{\Delta }}x}}\\ \left( {\frac{{\partial p}}{{\partial z}}} \right)_{j + 1/2} \approx \frac{{p_{i,j + 1} - p_{i,j - 1}}}{{{\mathrm{\Delta }}z}}\\ \left( {\frac{{\partial p}}{{\partial z}}} \right)_{j - 1/2} \approx \frac{{p_{i,j} - p_{i,j - 1}}}{{{\mathrm{\Delta }}z}}.\end{array}$$

Values of pore pressure in the element corners are approximated as the average of *p* in the four elements sharing the same node. For example, pore pressure at the $(i + 1/2,j + 1/2)$ node is given by

20 $$p_{i + 1/2,j + 1/2} = \frac{1}{4}\left( {p_{i,j} + p_{i,j + 1} + p_{i + 1,j} + p_{i + 1,j + 1}} \right)$$

in such a way that the integrals from (15) to (18) may be approximated as

21 $$\begin{array}{*{20}{l}} {{\int_{{\mathrm{\Delta }}V}} \frac{\partial }{{\partial x}}\left[ {K_{xx}\frac{{\partial p}}{{\partial x}}} \right]{\mathrm d}V} \hfill & \approx \hfill & {K_{xx_{i + 1/2,j}}\left( {p_{i + 1,j} - p_{i,j}} \right) -\, K_{xx_{i - 1/2,j}}\left( {p_{i,j} - p_{i - 1,j}} \right)} \hfill \\ {{\int_{{\mathrm{\Delta }}V}} \frac{\partial }{{\partial x}}\left[ {K_{xz}\frac{{\partial p}}{{\partial z}}} \right]{\mathrm d}V} \hfill & \approx \hfill & {\frac{1}{4}K_{xz_{i + 1/2,j}}\left( {p_{i,j + 1} + p_{i + 1,j + 1} + p_{i,j - 1} + p_{i + 1,j - 1}} \right)} \hfill \\ {} \hfill & {} \hfill & { - \frac{1}{4}K_{xz_{i - 1/2,j}}\left( {p_{i - 1,j + 1} + p_{i - 1,j - 1} + p_{i,j - 1} + p_{i,j + 1}} \right)} \hfill \\ {{\int_{{\mathrm{\Delta }}V}} \frac{\partial }{{\partial z}}\left[ {K_{zx}\frac{{\partial p}}{{\partial x}}} \right]{\mathrm d}V} \hfill & \approx \hfill & {\frac{1}{4}K_{zx_{i,j + 1/2}}\left( {p_{i - 1,j + 1} + p_{i - 1,j} + p_{i + 1,j + 1} + p_{i + 1,j}} \right)} \hfill \\ {} \hfill & {} \hfill & { - \frac{1}{4}K_{zx_{i,j - 1/2}}\left( {p_{i - 1,j} + p_{i - 1,j - 1} + p_{i + 1,j} + p_{i + 1,j - 1}} \right)} \hfill \\ {{\int_{{\mathrm{\Delta }}V}} \frac{\partial }{{\partial z}}\left[ {K_{zz}\frac{{\partial p}}{{\partial z}}} \right]{\mathrm d}V} \hfill & \approx \hfill & {K_{zz_{i,j + 1/2}}\left( {p_{i,j + 1} - p_{i,j}} \right) -\, K_{zz_{i,j - 1/2}}\left( {p_{i,j} - p_{i,j - 1}} \right).} \hfill \end{array}$$

Discretizing now the left-hand term of Eq. (14) in space, we obtain

$${\int_{\Delta V}} \frac{{\partial p\left( {x,z,t} \right)}}{{\partial t}}{\mathrm d}V = \frac{\partial }{{\partial t}}{\int_{z_{j - 1/2}}^{z_{j + 1/2}}} {\int_{x_{i - 1/2}}^{x_{i + 1/2}}} p\left( {x,z,t} \right){\mathrm d}x{\mathrm d}z = {\mathrm{\Delta }}x{\mathrm{\Delta }}z\frac{{\partial p_{i,j}}}{{\partial t}}.$$

Equating both discretized terms of Eq. (14), we finally get the discrete form of Eq. (13):

22 $$\begin{array}{*{20}{l}} {{\mathrm{\Delta }}x{\mathrm{\Delta }}z\frac{{\partial p_{i,j}}}{{\partial t}}} \hfill & \approx \hfill & {a_0p_{i,j} + a_1p_{i - 1,j + 1} + a_2p_{i,j + 1} + a_3p_{i + 1,j + 1} + a_4p_{i - 1,j}} \hfill \\ {} \hfill & {} \hfill & { + {\,}a_5p_{i + 1,j + 1} + a_6p_{i - 1,j - 1} + a_7p_{i,j - 1} + a_8p_{i + 1,j - 1}} \hfill \end{array}$$

where coefficients *a* are defined as

23 $$\begin{array}{*{20}{l}} {a_0} \hfill & = \hfill & { - \left( {K_{xx_{i + 1/2,j}} + K_{xx_{i - 1/2,j}} + K_{zz_{i,j + 1/2}} + K_{zz_{i,j - 1/2}}} \right)} \hfill \\ {a_1} \hfill & = \hfill & {\left( {\frac{{K_{zx_{i,j + 1/2}} -\, K_{xz_{i - 1/2,j}}}}{4}} \right)} \hfill \\ {a_2} \hfill & = \hfill & {\left( {\frac{{K_{xz_{i + 1/2,j}} -\, K_{xz_{i - 1/2,j}}}}{4} + K_{zz_{i,j + 1/2}}} \right)} \hfill \\ {a_3} \hfill & = \hfill & {\left( {\frac{{K_{xz_{i + 1/2,j}} +\, K_{zx_{i,j + 1/2}}}}{4}} \right)} \hfill \\ {a_4} \hfill & = \hfill & {\left( {K_{xx_{i - 1/2,j}} + \frac{{K_{zx_{i,j + 1/2}} -\, K_{zx_{i,j - 1/2}}}}{4}} \right)} \hfill \\ {a_5} \hfill & = \hfill & {\left( {K_{xx_{i + 1/2,j}} + \frac{{K_{zx_{i,j + 1/2}} -\, K_{zx_{i,j - 1/2}}}}{4}} \right)} \hfill \\ {a_6} \hfill & = \hfill & {- \left( {\frac{{K_{xz_{i - 1/2,j}} +\, K_{zx_{i,j - 1/2}}}}{4}} \right)} \hfill \\ {a_7} \hfill & = \hfill & {\left( {\frac{{K_{xz_{i + 1/2,j}} -\, K_{xz_{i - 1/2,j}}}}{4} + K_{zz_{i,j - 1/2}}} \right)} \hfill \\ {a_8} \hfill & = \hfill & {\left( {\frac{{K_{xz_{i + 1/2,j}} -\, K_{zx_{i,j - 1/2}}}}{4}} \right)} \hfill \end{array}$$

### Boundary conditions

To complete the scheme (22), we need update formulae also for the boundary points of the domain. This means setting values $p_{i,0},p_{i,N},p_{0,j}$, and $p_{N,j}$, where *N* represents the total number of elements per dimension, derived by taking the boundary conditions into account.

To this purpose, we impose Neumann conditions (i.e., no-flux) along the boundaries. This means that pore-pressure derivatives in the boundary-perpendicular directions are set to zero for all *t*. At boundary points, spatial derivatives are approximated with a second-order finite difference scheme considering ghost elements beyond the boundaries, so that the condition for any *j* at *i* = 0 (see Supplementary Figure 7) is given by

$$\frac{{\partial p_{0,j}}}{{\partial z}} \approx \frac{{p_{0,j} - p_{ - 1,j}}}{{{\mathrm{\Delta }}x}} = 0,$$

which implies that

24 $$p_{ - 1,j} = p_{0,j}.$$

By imposing this condition into scheme (22) for $p_{i,j} = p_{0,j}$, we obtain *p* values for the left-hand edge of the domain as

25 $$\begin{array}{*{20}{l}} {{\mathrm{\Delta }}x{\mathrm{\Delta }}z\frac{{\partial p_{0,j}}}{{\partial t}}} \hfill & \approx \hfill & {\left( {a_1 + a_2} \right)p_{0,j + 1} + a_3p_{1,j + 1} + \left( {a_4 + a_0} \right)p_{0,j}} \hfill \\ {} \hfill & {} \hfill & { +\, a_5p_{1,j} + \left( {a_6 + a_7} \right)p_{0,j - 1} + a_8p_{1,j - 1}.} \hfill \end{array}$$

Similarly, for the rest of the domain boundaries we obtain

26 $$\begin{array}{*{20}{l}} {{\mathrm{\Delta }}x{\mathrm{\Delta }}z\frac{{\partial p_{N,j}}}{{\partial t}}} \hfill & \approx \hfill & {a_1p_{N - 1,j + 1} + \left( {a_2 + a_3} \right)p_{N,j + 1} + a_4p_{N - 1,j} + \left( {a_5 + a_0} \right)p_{N,j}} \hfill \\ {} \hfill & {} \hfill & { +\, a_6p_{N - 1,j - 1} + \left( {a_7 + a_8} \right)p_{N,j - 1}} \hfill \\ {\Delta x\Delta z\frac{{\partial p_{i,0}}}{{\partial t}}} \hfill & \approx \hfill & {a_1p_{i - 1,1} + a_2p_{i,1} + a_3p_{i + 1,1} + \left( {a_4 + a_6} \right)p_{i - 1,0}} \hfill \\ {} \hfill & {} \hfill & { + \,\left( {a_5 + a_8} \right)p_{i + 1,0} + \left( {a_7 + a_0} \right)p_{i,1}} \hfill \\ {\Delta x\Delta z\frac{{\partial p_{i,N}}}{{\partial t}}} \hfill & \approx \hfill & {\left( {a_1 + a_4} \right)p_{i - 1,N} + \left( {a_2 + a_0} \right)p_{i,N} + \left( {a_3 + a_5} \right)p_{i + 1,N}} \hfill \\ {} \hfill & {} \hfill & { +\, a_6p_{i - 1,N - 1} + a_7p_{i,N - 1} + a_8p_{i + 1,N - 1}.} \hfill \end{array}$$

For the elements located at the domain corners we set both spatial derivatives to zero getting

27 $$ \begin{array}{l}\hskip -59pt {\mathrm{\Delta }}x{\mathrm{\Delta }}z\frac{{\partial p_{0,0}}}{{\partial t}} \approx \left( {a_1 + a_2} \right)p_{0,1} + a_3p_{1,1} + \left( {a_4 + a_6 + a_7 + a_0} \right)p_{0,0} + \left( {a_5 + a_8} \right)p_{1,0}\\ \hskip -35pt {\mathrm{\Delta }}x{\mathrm{\Delta }}z\frac{{\partial p_{N,0}}}{{\partial t}} \approx a_1p_{N - 1,1} + \left( {a_2 + a_3} \right)p_{N,1} + \left( {a_4 + a_6} \right)p_{N - 1,0} + \left( {a_5 + a_0 + a_7 + a_8} \right)p_{N,0}\\ \hskip -35pt {\mathrm{\Delta }}x{\mathrm{\Delta }}z\frac{{\partial p_{0,N}}}{{\partial t}} \approx \left( {a_1 + a_2 + a_4 + a_0} \right)p_{0,N} + \left( {a_3 + a_5} \right)p_{1,N} + \left( {a_6 + a_7} \right)p_{0,N - 1} + a_8p_{1,N - 1}\\ \hskip -4pt {\mathrm{\Delta }}x{\mathrm{\Delta }}z\frac{{\partial p_{N,N}}}{{\partial t}} \approx \left( {a_1 + a_4} \right)p_{N - 1,N} + \left( {a_2 + a_3 + a_5 + a_0} \right)p_{N,N} + a_6p_{N - 1,N - 1} + \left( {a_7 + a_8} \right)p_{N,N - 1},\end{array}$$

Expressions (22), (24), (25), and (26) represent a system of ordinary differential equations (ODEs) that may be expressed in the general form

28 $$\frac{{{\mathrm d}\underline p }}{{{\mathrm d}t}} \approx \frac{1}{{\Delta x\Delta z}}\underline {\underline A } \underline p ,$$

where $\underline p$ represents a $N \times N$ matrix while $\underline {\underline A }$ is a $N^2 \times N^2$ matrix. To solve system (28) in time, we used the MATLAB function edo15s, which yields robust solutions for stiff systems of ODEs by considering time-adaptive steps. We provide a verification of the FV method by comparing numerical predictions with analytical solutions in Supplementary Methods and in Supplementary Figure 8.

### Parametric analysis of the non-linear poroelastic equation

We performed a parametric analysis of Eq. (2) to identify the physical conditions leading to pore-pressure waves traveling with the observed RTM speeds. We considered values of $10^{ - 10} < k_0 < 10^{ - 16}$ m^2^ and pore pressure gradients (∇*p*_0_) ranging between 10^−1^ and 10^1^ MPa km^−1^. For *γ* parameter, we explored values around those derived from experimental tests, between 10^−8^ and 10^−5^ [Pa^−1^]^40^, and estimated porosities (*ϕ*) for the Cascadia subducted slab, between 1 and 2%^28^. For each combination of the parameters, we solved Eq. (2) assuming values for the remaining constants shown in Table 1 and track the pressure-wave front along 15 km for threshold values of *p* ranging between 1 and 10 kPa. Results obtained in the parametric analysis are summarized in Fig. 1c and Supplementary Figures 3 and 4, from which we determined the ranges of the model parameters (Table 2) producing pressure waves in the plate interface with speeds between 1 and 200 km/h.

### Code availability

The numerical codes we developed to produce the results of this study are available upon request to the corresponding author.

### Data availability

Data to produce the tectonic tremor catalog of this study is available upon request to the corresponding author.

## Electronic supplementary material

Supplementary Information
