# Supplementary material for: Rapid tremor migration and pore-pressure waves in subduction zones
Source: Nat Commun. 2018 Jul 24;9:2900. doi: 10.1038/s41467-018-05150-3 (PMC6057928; doi:10.1038/s41467-018-05150-3)
Supplement: Supplementary file 1 — Supplementary Information [file 41467_2018_5150_MOESM1_ESM.pdf]

Supplementary Information of

Rapid Tremor Migration and Pore-Pressure Waves in Subduction Zones

by Víctor M. Cruz-Atienza, Carlos Villafuerte and Harsha Bhat

Supplementary Note 1

In this supplement we present a verification exercise of the FV method where we compare numerical and analytical solutions for a given diffusion problem. We finally include some complementary results and figures properly referred in the main text of the manuscript.

Supplementary Methods

Verification of the 2D Finite Volume Method

To verify the implementation of our 2D FV solver for the diffusion equation (1), in this section we compare numerical predictions yielded by the FV approach with an analytical solution for a given problem with constant k in space and time. We had to make this assumption because, to our knowledge, there is no analytical solution for Equation (1) given the exponential form of $k(P_e)$ of the Equation (2), both of the main text. To approximate constant k in our approach we simply set parameter γ to zero.

For setting the pore pressure initial conditions of the problem, we chose de following function

$$p(x, z, t) = \exp(-2Kt)\cos(x)\cos(z), \tag{1}$$

which is a solution of Equation (1) of the main text for constant k and the no-flux Neumann boundary conditions introduced in the section of Methods [1]. From this function we can built the initial conditions for p simply by making t=0. As shown in Supplementary Figure 8a.

To complete the benchmark problem we assumed a constant diffusivity $K = \zeta k = 2m/s$ in the whole arbitrary domain defined by $[0, 2\pi]$ in each direction.

Supplementary Figure 8b presents the comparison of both solutions for $t = 0.5$ and 2.0 s. The red solid lines represent the analytical solution, while the blue dashed lines show the numerical solution yielded by our FV approach. Solutions correspond to p values along the diagonal dashed line of Supplementary Figure 8a. As a reference, the black curve shows the initial conditions along the same line. The excellent match between both predictions proves that the FV approach converges and produces accurate enough solutions.

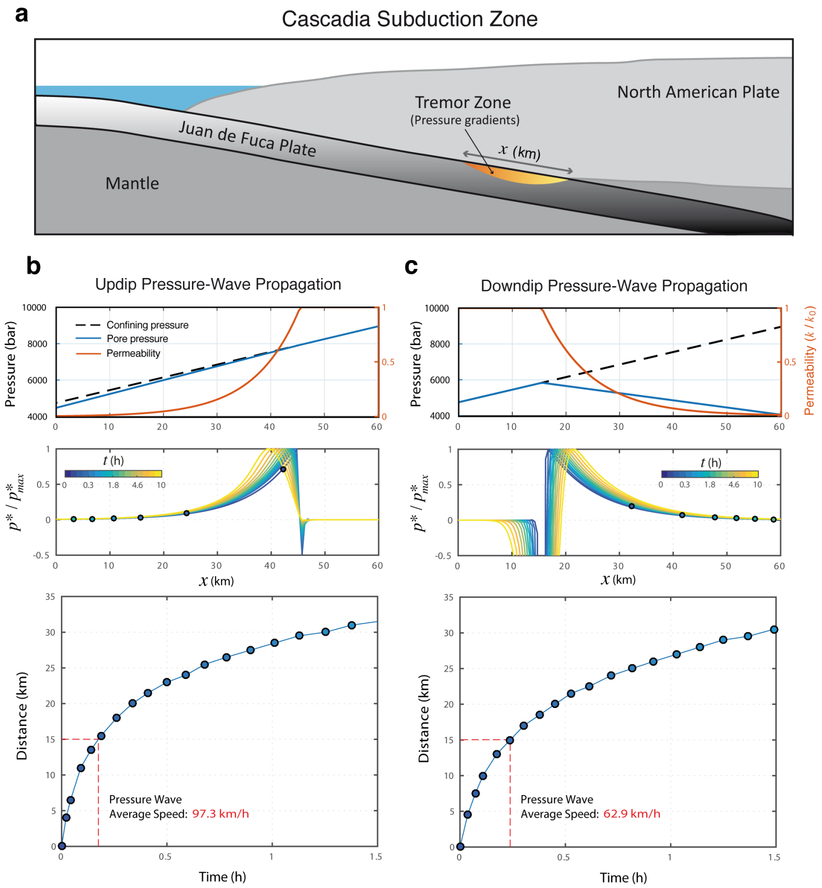

Supplementary Figure 1. RTM in Nankai and Cascadia exhibit a wide diversity of behaviors. For instance, most of the so-called streaks propagate in both the down-dip and up-dip opposite directions [2, 3, 4]. In contrast with the horizontal configuration of the slab in Guerrero (Figures 2 and 5a), in these subduction zones the OC sinks into the earth producing P_c gradients in the slip-parallel direction. This supplementary condition along with localized dehydration pulses and local variations of the plate-interface geometry may induce complex pore-pressure gradients likely to produce pressure waves in both directions within the active SSE front. In this figure we show the simulation results for two non-exhaustive examples considering the interface geometry in Cascadia, where pressure waves propagate in both opposite along-dip directions. (a) Cartoon showing the geometry of the Juan de Fuca plate under the continent. Gradient of gray colors illustrate the lithostatic pressure in the subducted slab, while the color gradient illustrate local pore pressure changes where RTMs are observed. (b) and (c) show, from top to bottom, the simulation initial conditions (for constant $k_0 = 1\text{e-}13\ \text{m}^2$), the pressure-waves propagation (where $p^* = p - p_0$) and the wave-front speed (p threshold of 3 kPa) for both updip and downdip propagation directions, respectively. The purpose of these simulations is just to illustrate that even in the presence of a downdip lithostatic pressure gradient, pressure-waves can propagate in both opposite directions with speeds similar to those observed in Cascadia. Although plausible, these Cascadia-like models should certainly be explored in future investigations.

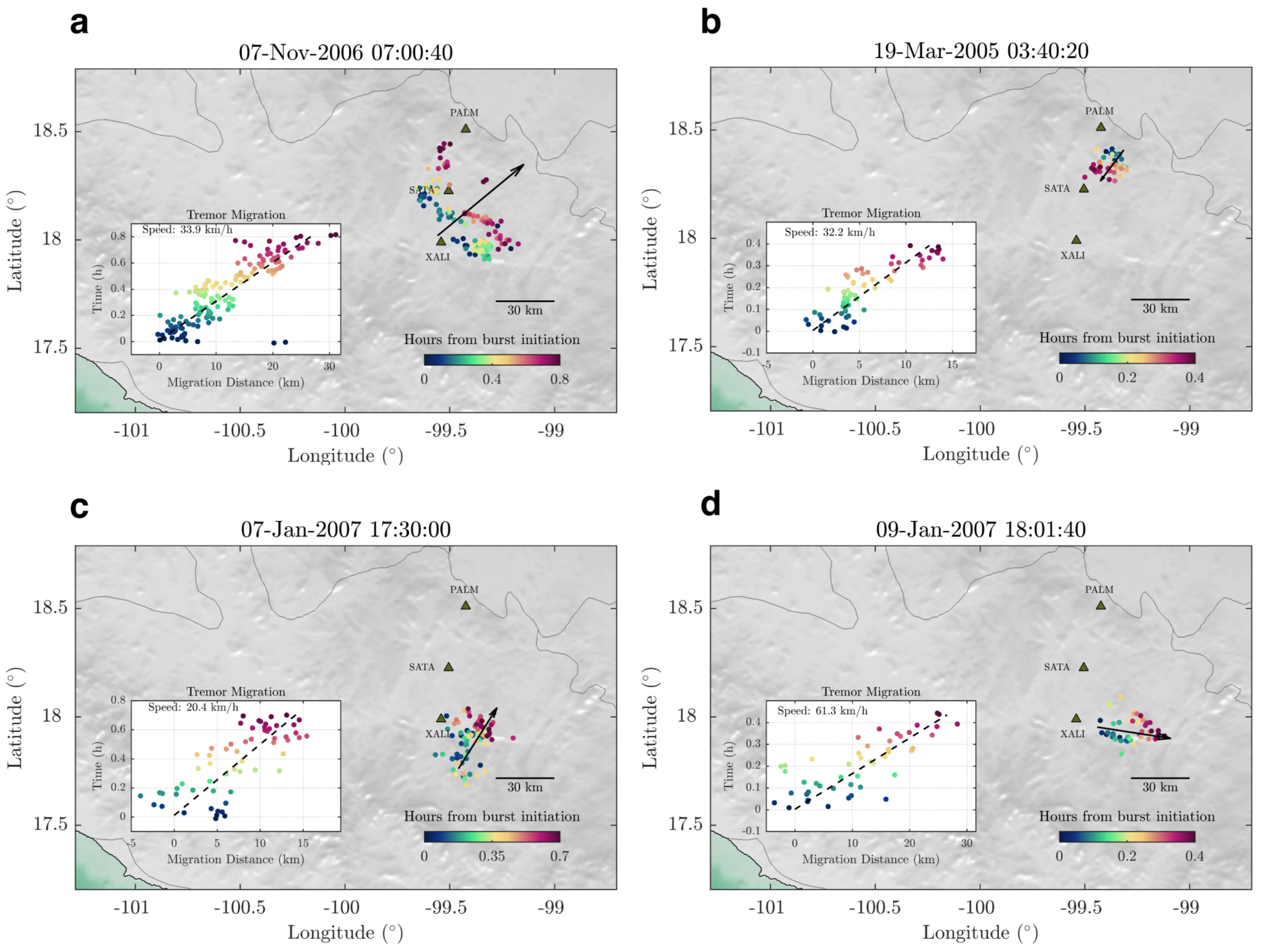

Supplementary Figure 2. Four examples of RTMs in Guerrero determined with the TREP method [5] (panels a to d). Tremor hypocenters were determined from one-minute moving windows with 20 s overlap. Hypocentral projections onto migration directions (black arrows) are show in the insets, where migration speeds are reported. The basemaps were created using SRTM15+ data.

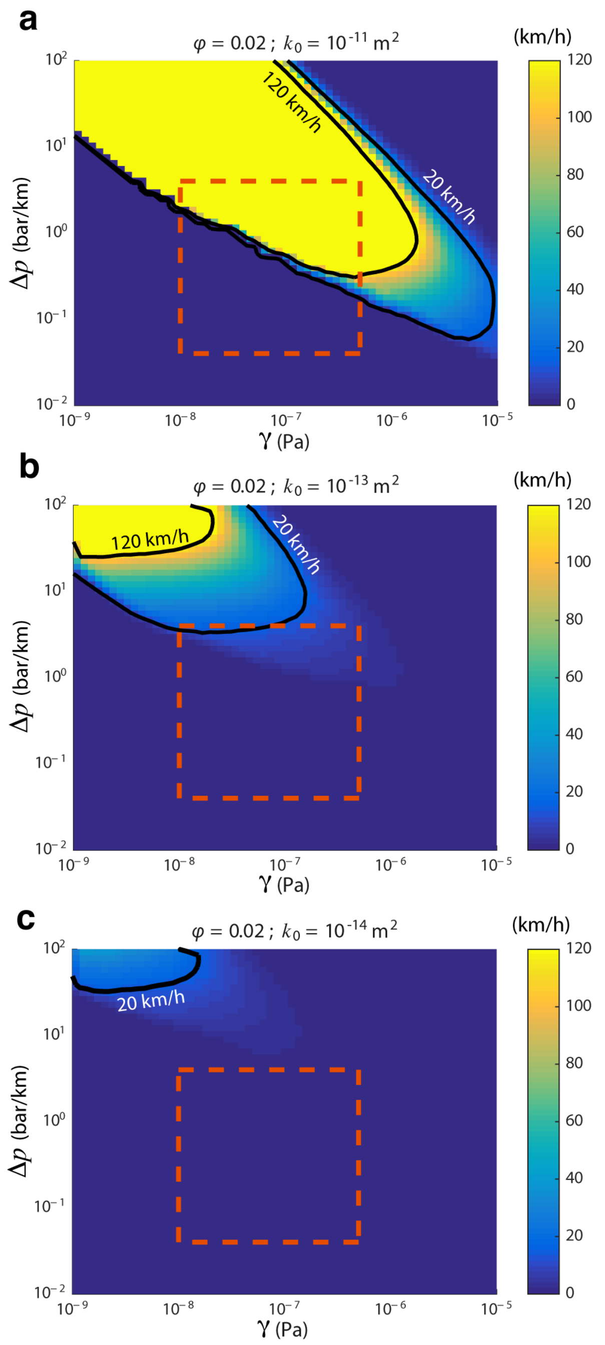

Supplementary Figure 3. Results from the parametric analysis of Equation 2 (main text) in terms of pressure-wave speeds (color shaded) for three different permeabilities k_0 (panels a to c) and a wave-front threshold of 3 kPa. Pressure-waves speed values between the black curves include those observed for RTMs in Guerrero. Red square delineates γ values observed in laboratory experiments [6] and the maximum pore-pressure gradient induced by the 2006 SSE (lower limit) and an arbitrary upper limit.

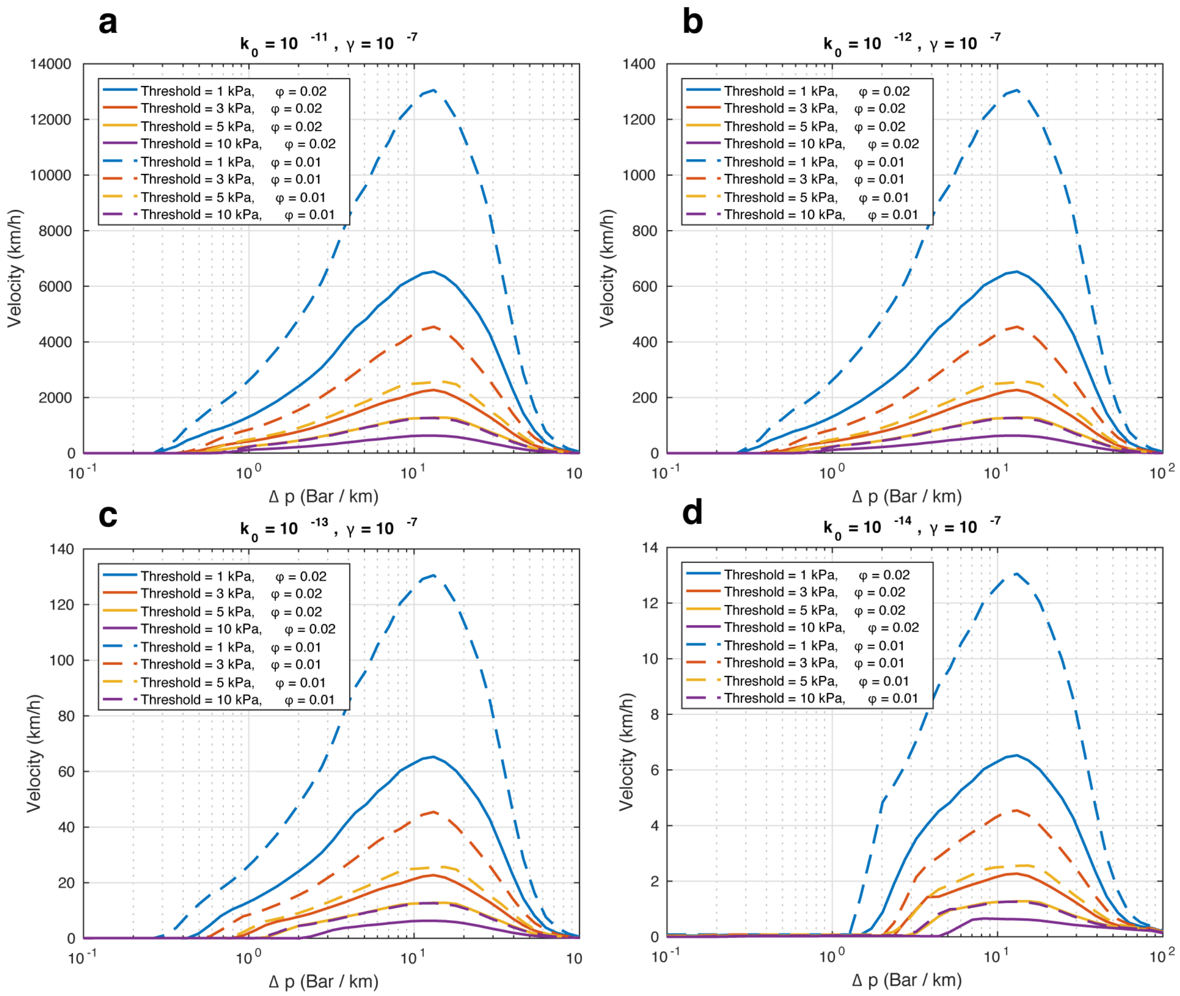

Supplementary Figure 4. Pore-pressure wave speeds (vertical axes) as a function of the pore-pressure gradient for four different permeabilities k_0 (panels a to d) detached from the parametric analysis of Equation 2 (main text). In each panel, we report wave speeds for four different values of the wave-front threshold and two values of porosity (i.e. 1 % and 2 %).

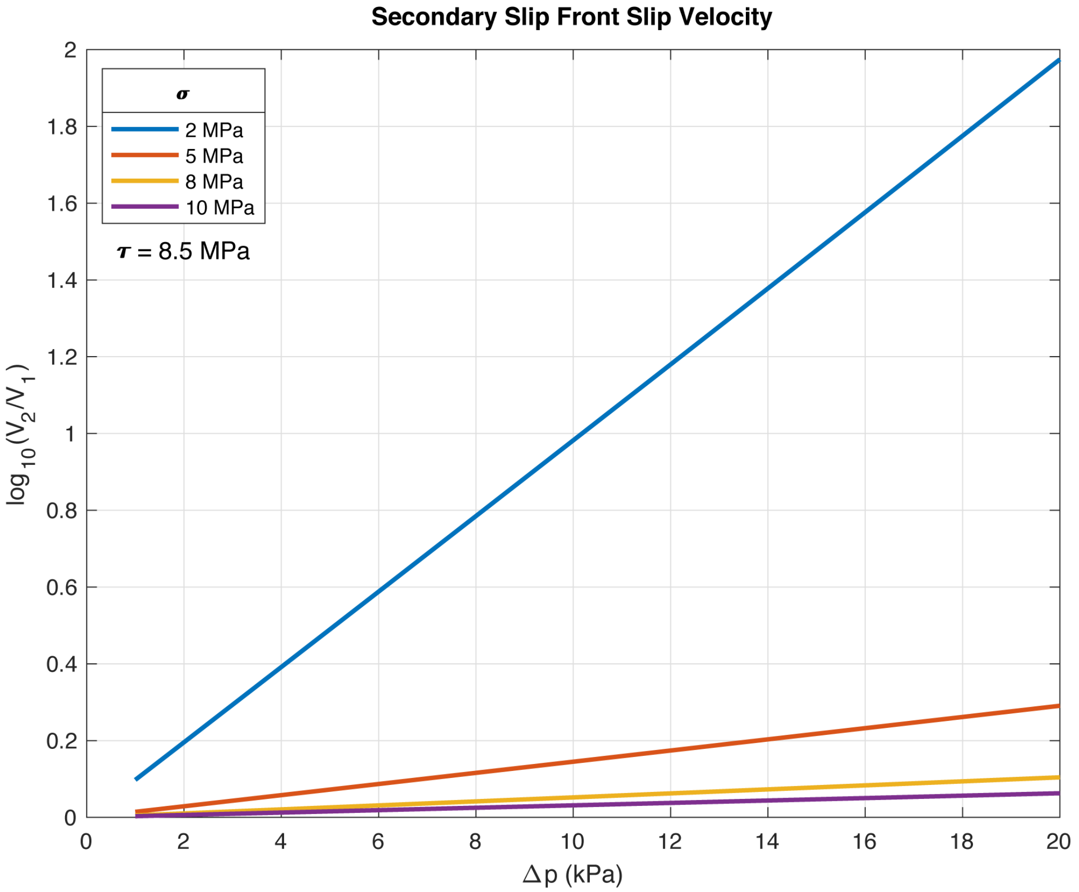

Supplementary Figure 5. Exponential growth of Secondary Slip Fronts (SSF) slip velocity (V_2) relative to the SSE slip velocity (V_1) under stable conditions in a R&S friction framework (i.e. velocity strengthening parameters) as a function of the pore pressure increment (Δp) in the fault. V_1 and V_2 represent the slip rates before and after the pore pressure increment has been applied, respectively, with constant shear (τ) and normal (σ) fault tractions. These curves have been generated using Equation 3 of the main text, which has been introduced by [7] for modeling SSEs.

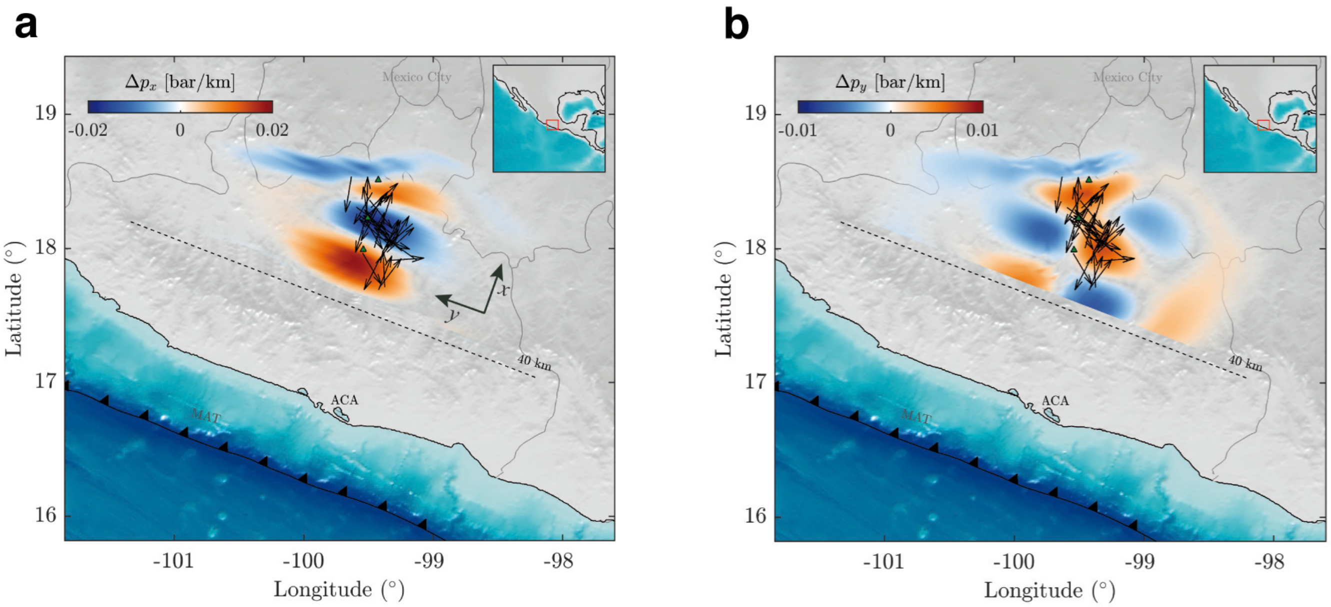

Supplementary Figure 6. Final pore-pressure gradient components (blue-red colors) induced by the 2006 SSE in the along-dip (a) and trench-parallel (b) directions (see main text). Blue colors indicate pressure reduction away from the trench in (a), and along the northwest trench-parallel direction in (b). Black arrows show the locations and propagation directions of the 54 RTMs found in the catalog. To calculate the gradient we simply estimated the change in p from the change in P_c assuming undrained conditions with a Skempton coefficient $B = 0.8$ (i.e. $p = B * Pc$) [8]. Two things stand out from the left figure: (1) most of RTMs in the down-dip direction lie within the minimum of the p gradient (blue colors), and (2) the minimum p gradient indicates a pore-pressure reduction with distance from the trench (i.e. in the RTM direction). This seems consistent with Equation 2, which predicts the propagation of pore-pressure waves towards depressurised regions (Figures 1a and S3). However, the maximum gradient values induced by the SSE (0.02 bar/km) are significantly smaller that those required to produce waves with the expected speeds (Table S2). Although the residual strain field from past SSEs may probably lead to larger pressure gradients, an additional preexistent gradient seems necessary in Guerrero to meet the conditions for rapid pressure-waves propagation. The basemaps and inset maps were created using SRTM15+ data.

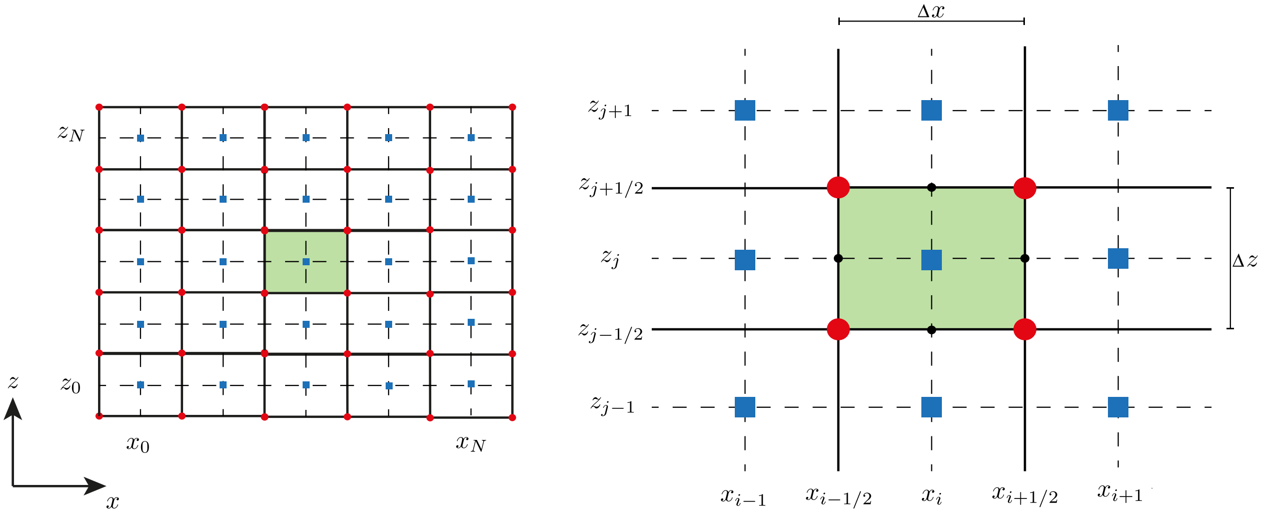

Supplementary Figure 7. Discretization of the 2D domain and representation of a cell volume $\triangle V$ (green cell). Values of the cell volumes are represented by blue squares in the center of each cell and the corner values by red circles. The black dots represent values over the faces of each cell. For this example $N = M$.

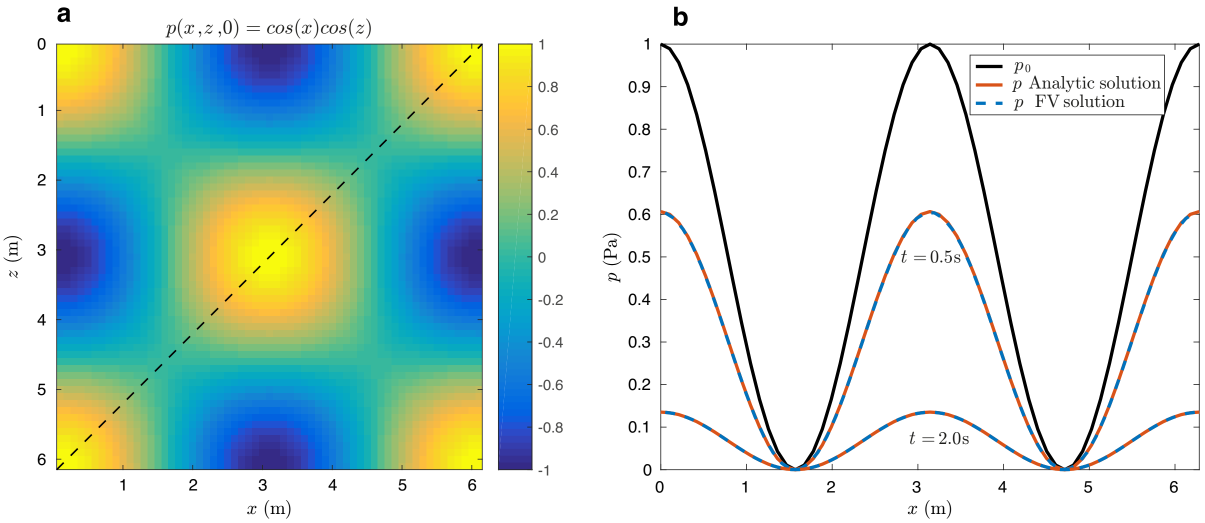

Supplementary Figure 8. (a) Initial conditions for p in 2D given by Equation 3 for $t = 0$.(b) Verification between the analytical (red) and the FV (dashed blue) solution over a diagonal section (black dashed line in a) for two different times at 0.5 and 2.0 seconds over the section. The black curve represent the initial condition of p over the dashed diagonal in a).

Supplementary References

[1] Peter V O′neil. *Advanced engineering mathematics*. Cengage learning, 2011.

[2] David R Shelly, Gregory C Beroza, and Satoshi Ide. Complex evolution of transient slip derived from precise tremor locations in western shikoku, japan. *Geochemistry, Geophysics, Geosystems*, 8(10), 2007.

[3] Abhijit Ghosh, John E Vidale, Justin R Sweet, Kenneth C Creager, Aaron G Wech, Heidi Houston, and Emily E Brodsky. Rapid, continuous streaking of tremor in cascadia. *Geochemistry, Geophysics, Geosystems*, 11(12), 2010.

[4] Quentin Bletery, Amanda M Thomas, Jessica C Hawthorne, Robert M Skarbek, Alan W Rempel, and Randy D Krogstad. Characteristics of secondary slip fronts associated with slow earthquakes in cascadia. *Earth and Planetary Science Letters*, 463:212–220, 2017.

[5] Víctor M Cruz-Atienza, Allen Husker, Denis Legrand, Emmanuel Caballero, and Vladimir Kostoglodov. Nonvolcanic tremor locations and mechanisms in guerrero, mexico, from energy-based and particle motion polarization analysis. *Journal of Geophysical Research: Solid Earth*, 120(1):275–289, 2015.

[6] James P Evans, Craig B Forster, and James V Goddard. Permeability of fault-related rocks, and implications for hydraulic structure of fault zones. *Journal of Structural Geology*, 19(11):1393–1404, 1997.

[7] Yajing Liu and James R Rice. Spontaneous and triggered aseismic deformation transients in a subduction fault model. *Journal of Geophysical Research: Solid Earth*, 112(B9), 2007.

[8] Carlos Villafuerte and Víctor M Cruz-Atienza. Insights into the causal relationship between slow slip and tectonic tremor in guerrero, mexico. *Journal of Geophysical Research: Solid Earth*, 122(8):6642–6656, 2017.
